# Clinical and Genetic Heterogeneity in Six Tunisian Families With Horizontal Gaze Palsy With Progressive Scoliosis: A Retrospective Study of 13 Cases

**DOI:** 10.3389/fped.2020.00172

**Published:** 2020-04-16

**Authors:** Sami Bouchoucha, Asma Chikhaoui, Dorra Najjar, Hamza Dallali, Maleke Khammessi, Sonia Abdelhak, Nabil Nessibe, Mohammad Shboul, Susanne G. Kircher, Ali Al Kaissi, Houda Yacoub-Youssef

**Affiliations:** ^1^Laboratoire de Génomique Biomédicale et Oncogénétique, LR16IPT05, Institut Pasteur de Tunis, Université Tunis El Manar, Tunis, Tunisia; ^2^Service Orthopédie, Hôpital d'enfant Béchir Hamza, Tunis, Tunisia; ^3^Department of Medical Laboratory Sciences, Jordan University of Science and Technology, Irbid, Jordan; ^4^Institute of Medical Chemistry, Medical University of Vienna, Vienna, Austria; ^5^First Medical Department, Ludwig Boltzmann Institute of Osteology, Hanusch Hospital of WGKK, AUVA Trauma Centre Meidling, Hanusch Hospital, Vienna, Austria; ^6^Pediatric Department, Orthopaedic Hospital of Speising, Vienna, Austria

**Keywords:** horizontal gaze palsy, ROBO3, orthopedic disorders, scoliosis, consanguinity

## Abstract

**Background:** Horizontal Gaze Palsy with Progressive Scoliosis (HGPPS) is a rare autosomal recessive congenital disorder characterized by the absence of conjugate horizontal eye movements, and progressive debilitating scoliosis during childhood and adolescence. HGPPS is associated with mutations of the *ROBO3* gene. In this study, the objective is to identify pathogenic variants in a cohort of Tunisian patients with HGPPS and to further define *ROBO3* genotype-phenotype correlations.

**Methods:** Thirteen Tunisian patients from six unrelated consanguineous families all manifesting HGPPS were genetically investigated. We searched for the causative variants for HGPPS using classical Sanger and whole exome sequencing.

**Results:** Four distinct homozygous mutations were identified in *ROBO3* gene. Two of these were newly identified homozygous and non-synonymous mutations, causing effectively damage to the protein by *in silico* analysis. The other two mutations were previously reported in Tunisian patients with HGPPS. Mutations were validated by Sanger sequencing in parents and affected individuals.

**Conclusion:** To the best of our knowledge, this is the largest ever reported cohort on families with HGPPS in whom *ROBO3* mutations were identified. These molecular findings have expanded our knowledge of the *ROBO3* mutational spectrum. The relevance of our current study is two-fold; first to assist proper management of the scoliosis and second to protect families at risk.

## Introduction

Aberrant axon connectivity in human results in many rare genetic disorders including corpus callosum agenesis, L1 syndrome, Joubert syndrome, Kallmann syndrome, Duane retraction syndrome, and Horizontal Gaze Palsy with Progressive Scoliosis.

Horizontal Gaze Palsy with Progressive Scoliosis (HGPPS; MIM 607313), first described in 1970 (1), is a rare autosomal recessive disorder belonging to a group of disorders known as congenital cranial dysinnervation disorders (CCDDs). It results from errors in cranial nuclear development and dysinnervation of the ocular and facial muscles (2–4). Affected individuals with HGPPS are born with restricted horizontal gaze with progressive scoliosis often occurring during infancy or early childhood (5, 6). HGPPS is caused by mutations in the *ROBO3* gene (MIM 607313) mapped to chromosome 11q23-25, consisting of 28 exons, and encoding a transmembrane receptor called roundabout homolog 3 protein (*ROBO3*) of 1384 amino acids. *ROBO3* is composed of an extracellular domain with five Ig-like and an intracellular domain, three fibronectin-like extracellular motifs, and three cytoplasmic signaling motifs (7). *ROBO3* plays a crucial role in commissural axon guidance and regulating midline axon crossing in vertebrates during embryonic development (8).

To date, 39 different variations have been described in the *ROBO3* gene associated with HGPPS phenotype (9). Four out of these 39 variations were identified in Tunisian patients (10). Many other patients remain clinically and genetically underdiagnosed.

Whole exome sequencing (WES) has become an important diagnostic tool that allows the investigations of thousands of genes simultaneously. In this study, we applied Sanger sequencing and WES to identify the causative variants in our Tunisian patients with HGPPS.

## Materials and Methods

### Ethical Approval

The families reported in this study provided informed signed consent for their involvement in this research, which was led according to Helsinki declaration and approved by the institutional review ethical committee of Institut Pasteur de Tunis (approval number 2018/27/E/HEBH/V2).

### Subjects

This is a retrospective study conducted between 2015 and 2019 in the Children's Hospital “Béchir Hamza.” Thirteen patients with clinical features suggestive of HGPPS were enrolled for clinical and radiological examination. After informed consent of legal tutors, the referral doctor examined the children. Upon examination, data including age, sex, Cobb angle of the spinal deformity and treatments of the scoliosis were collected. We also used a standardized questionnaire to inquire genetic history to draw familial pedigrees.

### DNA Extraction

Genomic DNA was manually extracted from peripheral blood collected in EDTA tubes using salting-out method. DNA quality was measured on a Nanodrop Spectrophotometer (Thermo Scientific, Wilmington, USA).

### Sanger Sequencing

Molecular investigation for recurrent mutations associated with HGPPS phenotype in Tunisian population, found previously was performed for exon 2, 4, 9, and 10 using Sanger sequencing. Primers were ordered as those used in the study done by Volk et al. (11). PCR products were directly sequenced using ABI Prism 3130 sequencer (Applied Biosystems, Foster City, CA, U.S.A.).

### Whole Exome Sequencing and Bioinformatics Analysis

Three Patients underwent whole exome sequencing (WES). Libraries were pooled together and reads were sequenced on the IlluminaNovoseq 6000 platform (Illumina, San Diego, CA, USA). Sequence quality control was done with FastQC (https://www.bioinformatics.babraham.ac.uk/projects/fastqc/), Read mapping to Genome Reference Consortium Human Build 37 (GRCh37) was performed with Burrows-Wheeler alignment (12). SNP and INDEL calling, together with advanced variant annotation were done using GATK and annotation was done with ANNOVAR.WES revealed 52599 homozygous variants within the capture regions in average for each patient. Variants were further filtered using VarAFT according to population frequency of variants that was determined using databases such as 1000 Genomes Project and Exome Aggregation Consortium (ExAC) v0.3. Only novel and rare variants were included in these analyses, defining rare as minor allele frequency (MAF) <0.5%. They were subsequently filtered based on their type and genomic localization. Pathogenicity assessment was done according to ACMG guidelines and using *in silico* prediction tools UMD Predictor (http://umd-predictor.eu/), SIFT (http://sift.jcvi.org/), PolyPhen-2 (http://genetics.bwh.harvard.edu/pph2/), and Mutation Taster (http://www.mutationtaster.org/).

## Results

### Clinical and Radiological Results

A detailed and complete clinical description of the largest cohort of HGPPS in North Africa is summarized in Table 1.

**Table 1:** Clinical features summary for the six families.

**Families/patients**		**Distortion discovery age (years)**	**Circumstances of discovery**	**Associated torticollis**	**Type of scoliosis**	**Cobb angle at the time of diagnosis**	**Orthopedic treatment**	**Surgical treatment**	**Age at last decline**
Family 1	Patient 1	Early childhood	Scoliosis	No	Right thoracic	28°	Bracing	No	10–15
	Patient 2	Early childhood	Torticollis	Yes	Right thoraco lumbar	10°	No	No	2–5
Family 2	Patient 1	Early childhood	Torticollis	Yes	Left thoracic	30°	Casting and bracing	Single traditional growing rod	10–15
	Patient 2	Early childhood	Torticollis	Yes	Left thoracic	15°	No	No	5–10
	Patient 3	Early childhood	Systematic screening	Yes	Left thoracic	10°	Bracing	No	2–5
Family 3	Patient 1	Early childhood	Scoliosis	No	Left thoracic	120°	Bracing	Anterior and posterior spinal fusion	20–25
	Patient 2	Early childhood	Scoliosis	No	Right thoracic	120°	Bracing	Anterior and posterior spinal fusion	20–25
	Patient 3	Early childhood	Scoliosis	No	Right thoracic	120°	Bracing	Waiting for surgical treatment	30–35
Family 4	Patient 1	Early childhood	Scoliosis	No	Left thoracic	-	Casting and bracing	Posterior spinal fusion	20–25
	Patient 2	Early childhood	Scoliosis	No	Right thoracolumbar	-	Casting and bracing	Anterior and posterior spinal fusion	15–20
Family 5	Patient	Early childhood	Scoliosis	Yes	Left thoracic	90°	No	Dual traditional growing rods	5–10
Family 6	Patient 1	Early childhood	Scoliosis	No	Right thoracic	90°	Bracing	Posterior spinal fusion	10–15
	Patient 2	Early childhood	Scoliosis	No	Left thoracic	90°	No	No	65–70

#### Family 1

**Figure 1:**
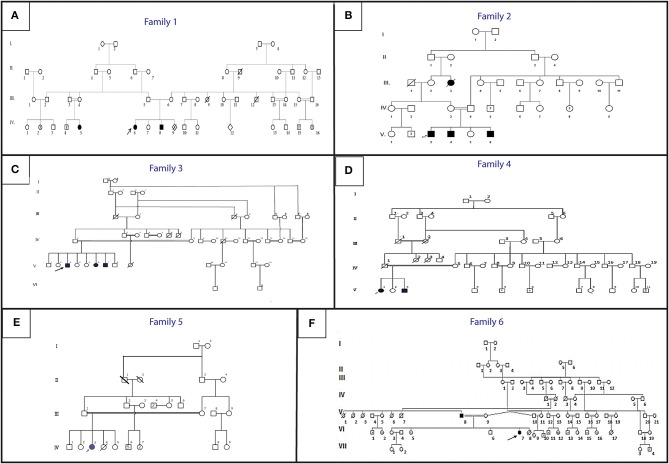
Pedigree describing affected HGPPS family's members. Filled symbols represent affected individuals, open symbols represent unaffected individuals. The probands are indicated with an arrow. **(A)** Family 1, **(B)** Family 2, **(C)** Family 3, **(D)** Family 4, **(E)** Family 5, **(F)** Family 6.

An 11-year-old ethnic Tunisian girl (V-9) born from first-degree consanguineous family and her 2 year-old brother (V-6) attended the Children's Hospital “Béchir Hamza” for pediatric examination, and concomitant genetic counseling with their parents who come from the central region of Tunisia (Figure 1A). The patient (V-9) had been seen for the first time for a right thoracic scoliosis with a Cobb angle of 28°. The spinal deformity was observed at the age of 3 years. On physical examination, she had a squint and conjugate horizontal gaze palsy consistent with the diagnosis of HGPPS. Bracing treatment was started and the spinal deformity remained stable after a 3-year follow up. Further investigations showed no intellectual disability nor morphological abnormalities in her parents.

Her younger brother (patient V-6) had also clinically discernible features of a squint with a conjugate gaze palsy and a right torticollis. The spinal radiograph showed a mild right thoracolombar scoliosis with a Cobb angle of 10°. After 1-year follow up without treatment, the scoliosis remained stable with the same Cobb angle.

During the genetic inquiry, our attention was drawn to another family member (V1) with similar clinical manifestations. Unfortunately, she was out of reach.

#### Family 2

Three siblings (V-3, V-4, and V-6) consulted, respectively, at the age of 12, 9, and 1 (Figure 1B). They were born to healthy parents originating from the northwest region of Tunisia after natural conception.

In this family, the 3 brothers exhibited a clinical phenotype of scoliosis with variable severity.

The older brother (V-3) was first seen when he was 1 year old for a squint associated with a left torticollis, which was at first mistaken with a muscular torticollis. At the age of three a left thoracic scoliosis with a Cobb angle of 30° associated with a conjugate horizontal gaze palsy was observed. We started a treatment with serial casting and bracing which failed to stop the progression of the deformity that reached a Cobb angle of 60°. This evolution prompted a surgical treatment with a single traditional growing rod. The patient is now 12 years old and the deformity is stable. The torticollis progressively disappeared and the patient developed a nystagmus.

**Figure 2:**
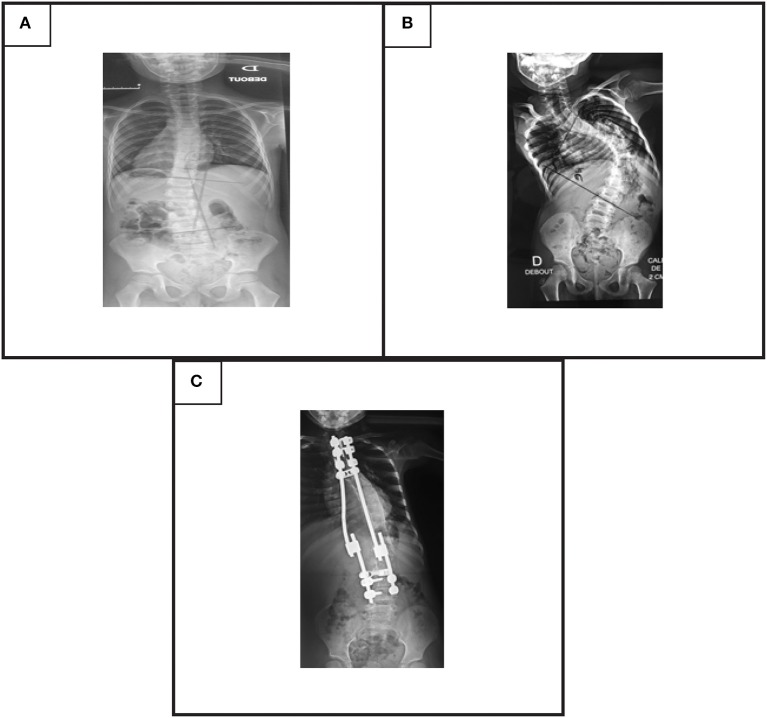
Postero-anterior view of X-ray showing scoliosis: **(A)** mild double major scoliosis stable without treatment in patient V4 of family 2, **(B)** severe thoracic scoliosis with a 94° Cobb's angle in patient IV3 of family 5, **(C)** 2 years after surgical correction with dual traditional growing rods in patient IV3 of family 5.

The second patient (V-4) was seen at the age of 8 months for a torticollis. A spinal X-ray showed a mild double major scoliosis. A yearly observation was established. The child is now 9 years old. The scoliosis is stable without treatment and its Cobb angle remains at 15° (Figure 2). This patient developed also a severe nystagmus.

The youngest brother (V-6) was diagnosed for the first time at the age of 8 months. He had a right torticollis and a squint with a conjugate horizontal gaze palsy. A left thoracic scoliosis with a Cobb angle of 10° was also discovered. Within 1 year, the deformity worsened which led to an orthopedic treatment with casting and bracing.

#### Family 3

Three siblings were treated with a brace in another institution for an infantile scoliosis. The horizontal gaze palsy was not diagnosed at that time. We do not have enough details about the previous orthopedic treatment. All three patients had a severe spinal deformity when first seen in our institution.

Patient V-3 consulted for the first time at the age of 16. He is the sixth child of healthy first cousin parents originating from west central Tunisia (Figure 1C). He presented symptoms during early childhood. He had a left thoracic scoliosis with a Cobb angle of 120°, which has been treated by an anterior and posterior fusion after halo gravity traction. His 21-year-old sister (V-6) had a right thoracic scoliosis with a Cobb angle of 120°, which has also been corrected the same way. The older brother (V-3) who is now 32, has a 120° right thoracic scoliosis and is waiting for surgical treatment.

#### Family 4

Patient V-1, a 21-year-old female and her 17-year-old brother (V3) (Figure 1D) are born from consanguineous parents originating from North-East Tunisia. Both patients had spinal fusion for scoliosis with horizontal gaze palsy.

The spinal deformity was discovered when they were 1.5 year old. A treatment was started with casting and bracing but the deformity worsened. The girl had a posterior spinal fusion at the age of 13 for a left thoracic scoliosis and the boy had an anterior and posterior spinal fusion for a right thoracolombar deformity.

#### Family 5

Patient IV-3 (Figure 1E) is a 7- year-old female born from first-degree consanguineous marriage originating from the North of Tunisia. This patient with a 94° left thoracic scoliosis with a torticollis, a squint and a conjugate horizontal gaze palsy. The deformity was seen several years earlier but no treatment was performed. A treatment with traditional dual growing rods was started. After a 2-year follow up and two distractions, the deformity became stable with a Cobb angle of 40° (Figures 2B,C).

#### Family 6

The patients are a father and his daughter originating from a consanguineous family from the Northern region of Tunisia (Figure 1F). The 14- year-old patient (IV-7) had a posterior spinal fusion for a right thoracic scoliosis with a Cobb angle of 90°. Her father aged 70 also has a left thoracic scoliosis of 90°, which was never treated. In both cases, the deformity started in early childhood.

### Genetic Findings

**Figure 3:**
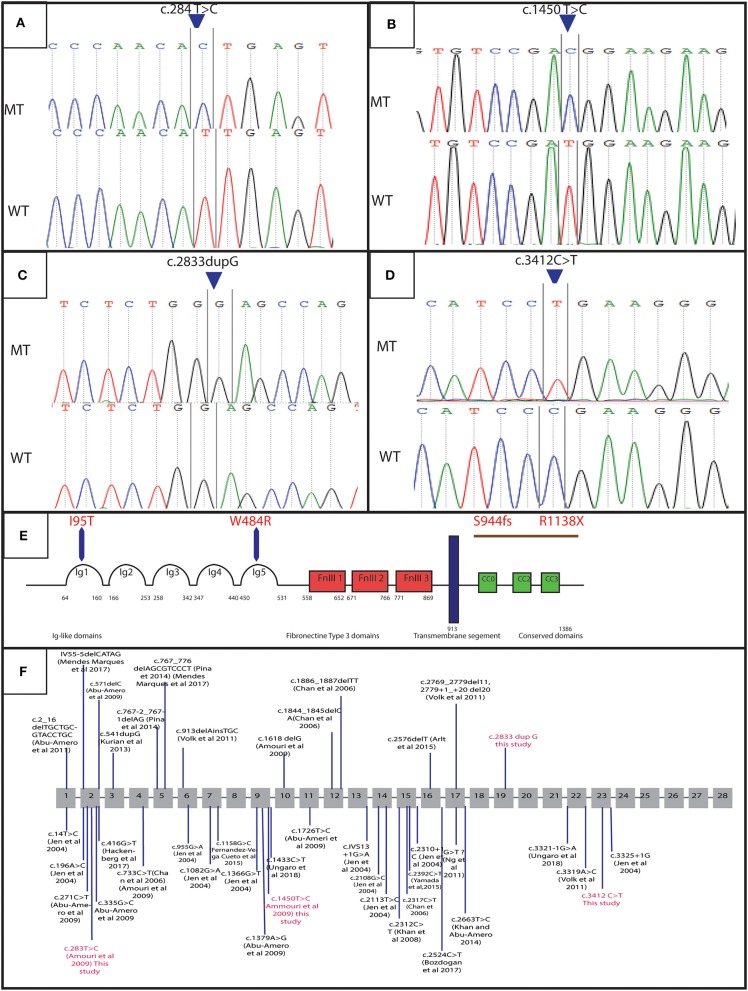
Genetic results: electropherograms showing the mutations in *ROBO3* gene: **(A)** exon 4 at a homozygous state in the patients of family 4 (c.284 T>C p. I95T), **(B)** exon 9 at a homozygous state in the patients of family 2 (c.1450 T>C p.W484R), **(C)** exon 19 in patients of family 1 and 3 (c.2833dupG:p.S944fs), **(D)** Exon 23 for patients of family 5 and 6 (c.3412C>T:p.R1138X), **(E)** protein representation, **(F)** linear map of the reported variations in *ROBO3*gene. Above (small additions and deletions) below point mutations. Variations in red are for mutations detected in this study.

In order to determine the genetic cause of the HGPPS in these patients, we performed Sanger sequencing and next-generation exome sequencing. Direct sequencing of exon 2, 4, 9, and 10 of *ROBO3* gene revealed two recurrent missense mutations in 5 patients from two unrelated families (10). Three probands (V-3, V-4, and V-6) of family 4 harbored the c.284T>C; p.I95T mutation in exon 4 and two affected children (V-1 and V-3) of family 2 carry the c.1450T>C; p.W484R mutation in exon 9 (Figures 3A,B). Parents were heterozygous carriers. No mutation was identified in the rest of families; therefore, samples underwent whole exome sequencing (WES).

WES analysis revealed a novel homozygous nonsense mutation in exon 23 of *ROBO3 gene* (c.3412C>T; p.R1138X) in both family 5 (patient IV-3) and 6 (patient IV-7) (Figures 3C,D). Sanger sequencing confirmed the heterozygous form of this mutation in both parents.

Another novel homozygous base pair duplication in exon 19 (c.2833dupG; p.S944fs) was also identified in family 1 (patient V-6 and V-9) and family 3 (patient V-3 and V-6). The c.2833dupG is predicted to disrupt the reading frame, causing a frameshift in the preceding sequence, resulting in a premature codon stop in the amino acid 985. Using online prediction programs such as Mutation tester, SIFT and POLYPhen, this variant is confirmed to be pathogenic, in cause of the disease. Both parents were heterozygous for the mutation.

We also identified a homozygous missense mutation (c.232G>C; p.G78R) in exon 4 of *CABP4* gene (MIM 608965) in patient V-9 of the family 1(data not shown). Mutations in this gene have been reported as responsible for a rare disorder phenotype cone-rod synaptic disorder or night blindness (MIM 610427) (13). Further clinical diagnosis showed that pathological effect was absent in the patient.

## Discussion

Congenital cranial dysinnervation disorders (CCDDs), are a group of anomalies, which describe abnormal movements of the eyes. Among these diseases, congenital fibrosis of the extraocular muscles (CFEOM), Duane Retraction Syndrome (DURS), and Congenital Horizontal Gaze Palsy with Progressive Scoliosis (HGPPS) share similarities in phenotype.

The first description of HGPPS in a family with scoliosis associated with progressive external ophthalmoplegia was reported by Dretakis (14). Dretakis and Kondoyannis later described 5 other cases of early onset scoliosis associated with horizontal gaze palsy in 2 consanguineous Greek families (1). Several papers have been published since then, describing the clinical spectrum of HGPPS. In this study, we report detailed clinical and radiological features of a large cohort of patients with HGPPS, the evolution and management of spinal deformity as well as the genetic diagnosis.

The spinal deformity was observed in most patients at <3 years of age and three of them before the age of one. Neonatal diagnosis has been reported in some cases mostly due to ophtalmologic abnormalities (11, 15, 16). Neonatal scoliosis has also been reported (17). The scoliosis is often observed in early childhood or even in the first year of life with an average age at diagnosis of 4.3 year (16, 18–21), and is rarely reported in adolescence (22).

Left and right thoracic scoliosis have been reported in many cases with HGPPS (16, 20, 21, 23). In our patients spinal X-rays showed more often left thoracic scoliosis. Most spinal deformities were severe in our patients with a Cobb angle at skeletal maturity reaching more than 90° in most cases and often more than 120°. This severity is a common feature of scoliosis in HGPPS (20, 21, 23). Less severe deformities can be seen either without treatment or after orthopedic treatment with no need of surgical correction (11, 19, 24–26). We were able to follow most of our patients over a long period since early childhood. Only one of them had a mild deformity with a Cobb angle of 15° that remained stable without treatment after several years. However, his oldest brother had a scoliosis that worsened rapidly despite orthopedic treatment with serial casting and brace and required surgical treatment with growing rods at age 6, highlighting the possible intra-familial variability in the severity of the scoliosis. This variability has also been reported by Ungaro et al. (27). However, the scoliosis is most often very highly progressive during childhood (16, 19, 21, 23). It is therefore, often necessary to treat the spinal deformity in young children with growth-friendly surgery if the scoliosis is severe, either at diagnosis or after a failed attempt of orthotic treatment.

Torticollis, which has been reported in several patients with HGPPS, can also be a reason for discovery (18, 28). In this study, it was observed in young patients but it seems that it resolved over time.

Patients with HGPPS have characteristic lesions of central nervous system that are clearly visible on magnetic resonance imaging (MRI) (5). These lesions are a hypoplastic butterfly configuration of the medulla oblongata, with the absence of facial colliculi and hypoplastic pons. The pons is split into two halves by a midsagittal cleft extending from the floor of the fourth ventricle. This creates a “tent shaped” fourth ventricle that can be seen on axial images. On sagittal images, a depression of the fourth ventricle is noted. The pathogenesis of HGPPS involves decussation anomalies with uncrossed sensory and motor pathways at the level of the brainstem. This was suggested by several observations of hemiplegia ipsilateral to the cerebral lesion after a stroke in patients with HGPPS (17, 29, 30) as well as neurophysiologic observation when surgery of scoliosis was performed (20, 31). The decussation abnormality has also been seen by functional MRI (22, 32).

A Total of 39 different variations in the *ROBO3* gene associated with HGPPS phenotype has been described worldwide [Figure 3F; (9)]. In the Tunisian population, 10 cases have been previously investigated for HGPPS using linkage analysis and 4 different mutations in *ROBO3* were reported (10). Another previous study reported only clinical features associated with HGPPS in eight Tunisian patients (15).

In this article, we identified 4 distinct mutations in 13 patients with HGPPS from six unrelated consanguineous families. Two of these mutations are novel and two have already been reported in the Tunisian population. The recurrence of these mutations in the same ethnic group suggests that they could represent founder alleles in the Tunisian population.

The use of next generation sequencing technique allows us to identify two novel variations in *ROBO3* gene. According to the clinical phenotype in these patients, the two variations are likely loss-of-function alleles.

In family 1 and 3, we detected the c.2833dupG; in exon 19. This frameshift mutation is located at C-terminus of the cytoplasmic domain in *ROBO3* protein. It is predicted to result in the formation of an unstable mRNA transcript that is subsequently subjected to a nonsense-mediated mRNA decay (NMD), or the formation of a truncated protein (p.S944fs) that lacks 402 amino acids including the cytoplasmic signaling motif CC2 and CC3. The absence of the cytoplasmic domains could interfere with signaling pathway. Further work to explain the role of these domains in the *ROBO3* function and their implication in the disease is needed. Moreover, *in silico* analysis using online prediction programs showed that this mutation could disrupt the splice site.

In family 5 and 6, we identified a truncated mutation c.3412C>T; p.R1138X in exon 23 of *ROBO3*. This variant is located in the C-terminal region. It is predicted to induce a premature stop codon at residue 1138, associated with mRNA degradation via NMD or formation of a truncated protein that lacks 249 amino acids including the cytoplasmic signaling motif CC3 (Figure 3E). This domain might play an important role in the normal function of *ROBO3* protein (4). Therefore, the absence of CC3 could explain HGPPS phenotype in these patients. In this same exon 23, Jen et al. have identified a single base pair insertion mutation (c.3325+1G), which leads to premature protein termination (7). Similarly, Volk et al. also described a homozygous missense in two affected siblings located next to a splice donor site (c.3319A>C) resulting in skipping of exon 22 that encodes for the cytoplasmic motifs (11). These findings are strongly in favor of the pathogenic effect of the variation.

Interestingly, we also found a new variant c.232G>C, p.G78R in exon 4 in CABP4 gene in patient V-5 of family 1. This variant was predicted to be pathogenic by mutation taster and sift and could alter the splice site according to a human splicing finder prediction tool. Mutations in CABP4 are responsible for a rare congenital non-progressive cone-rod synaptic disorder (13). However, clinical examination of this patient rejects the plausible pathological effect of the variant. This suggests the importance of the association between computational algorithms and their relation to clinical examinations.

The fact that the frequency of consanguineous marriage in Arab countries including Tunisia is very high leads to an increase of the expression of autosomal recessive diseases (33–35). HGPPS seems to be another example, as all patients come from consanguineous marriage.

The genotype-phenotype correlation is still unclear in HGPPS, possibly because of the poor information about the role of various *ROBO3* domains. Compared with previous investigations, our results enlarge the mutational spectrum and suggest the impact of the cytoplasmic domains C2 and C3 in the emergence of the pathology. This hypothesis was also advocated by Ungaro et al. (9).

Our results added new variants to the mutational spectrum of HGPPS disease and highlighted the role of the high rate of consanguinity as a causation for accumulated handicapping and malformation.

The discovery of the genetic etiology of HGPPS disease in Tunisia will be helpful for a better and an earlier clinical management via following up of patient's scoliosis since birth in the family at risk.

## Data Availability Statement

The datasets generated for this study can be found in LGBMO_Server_IPT, and can be requested by email to the corresponding author.

## Ethics Statement

The families reported in this study provided informed signed consent for their involvement in this research, which was led according to the Helsinki declaration and approved by the institutional review ethical community of institute Pasteur de Tunis approval number (2018/27/E/HEBH/V2).

## Author Contributions

SB did the clinical investigation of patients and available family members with follow up, and drafted the clinical section of the manuscript. AC and SB did the genetic experiments, analysis and interpretation of data, and drafted the genetic section of the manuscript. DN, HD, and MK helped in analyzing WES data. SA contributed in the design of study. NN, MS, SK, AA, and HY-Y did Clinical advice and critical revision of the manuscript. HY-Y supervised and helped in the conception of the study.

### Conflict of Interest

The authors declare that the research was conducted in the absence of any commercial or financial relationships that could be construed as a potential conflict of interest.
